# The Complex Role of Regulatory T Cells in Immunity and Aging

**DOI:** 10.3389/fimmu.2020.616949

**Published:** 2021-01-27

**Authors:** Lourdes Rocamora-Reverte, Franz Leonard Melzer, Reinhard Würzner, Birgit Weinberger

**Affiliations:** ^1^ Department of Immunology, Institute for Biomedical Aging Research, University of Innsbruck, Innsbruck, Austria; ^2^ Institute of Hygiene & Medical Microbiology, Department of Hygiene, Microbiology and Public Health, Medical University Innsbruck, Innsbruck, Austria

**Keywords:** regulatory T cells, immune homeostasis, diversity, autoimmunity, inflammation, aging

## Abstract

The immune system is a tightly regulated network which allows the development of defense mechanisms against foreign antigens and tolerance toward self-antigens. Regulatory T cells (Treg) contribute to immune homeostasis by maintaining unresponsiveness to self-antigens and suppressing exaggerated immune responses. Dysregulation of any of these processes can lead to serious consequences. Classically, Treg cell functions have been described in CD4^+^ T cells, but other immune cells also harbour the capacity to modulate immune responses. Regulatory functions have been described for different CD8^+^ T cell subsets, as well as other T cells such as γδT cells or NKT cells. In this review we describe the diverse populations of Treg cells and their role in different scenarios. Special attention is paid to the aging process, which is characterized by an altered composition of immune cells. Treg cells can contribute to the development of various age-related diseases but they are poorly characterized in aged individuals. The huge diversity of cells that display immune modulatory functions and the lack of universal markers to identify Treg make the expanding field of Treg research complex and challenging. There are still many open questions that need to be answered to solve the enigma of regulatory T cells.

## Introduction

Immunological self-tolerance is the unresponsiveness of the adaptive immune system to self-antigens in primary lymphoid organs and further control of the activation, expansion and survival of self-reactive T and B cells in the periphery (1). The acquisition of immune tolerance is essential to avoid fighting one´s own cells and molecules. During thymic maturation of T cells somatic recombination leads to the expression of a distinct T cell receptor (TCR) on each individual T cell, which enables each T cell to recognize a specific antigen. The entirety of all T cell specificities is referred to as the repertoire. Binding of TCRs to self-peptide-loaded MHC molecules (self-pMHC) in the thymus leads to positive selection, and in a next step T cells binding self-pMHC with high affinity are eliminated by negative selection. Thymocytes can escape this clonal deletion by TCR gene rearrangement, which eventually changes the TCR affinity for self-pMHC. This central tolerance is not absolute as not all self-antigens are expressed in the thymus and due to the imperfect efficiency of the selection process. As a consequence, depletion of self-reactive T cells can also occur in the periphery and many self-reactive T cells can enter a state of unresponsiveness called “anergy” (peripheral tolerance) (2).

Some decades ago it was shown that animals harbor self-reactive T cells but also T cells that were able to suppress the autoimmunity caused by these cells (3, 4). These suppressor cells were later called regulatory T (Treg) cells and the surface molecule CD25 was identified as the first marker for this CD4^+^ T cell subset (5). It was not until 2003 that the transcription factor Forkhead box P3 (Foxp3) was described as a specific Treg marker by Rudensky’s laboratory. They discovered that Foxp3 deficient mice developed a lethal autoimmune syndrome and lacked CD4^+^ CD25^+^ cells (6). However, not all suppressor/regulatory T cells express the Foxp3 transcription factor, and many of those other populations have been described even before the Foxp3 expressing T cells. In this review, we will focus mainly on Foxp3 expressing Treg, their mechanisms of suppression and their role in different contexts. Nevertheless, a short description of Foxp3^-^ Treg cells is necessary to understand the complexity of the exquisitely regulated homeostasis of the immune system. Regulation of immune responses requires not only the control of autoreactive immune cells but also the termination of immune responses in order to avoid chronic activation of the immune system. It is important to understand how these mechanisms are regulated in order to modulate immune responses in different disease settings. For instance, we need to strengthen immune responses in chronic infection or cancer but, on the other hand, immune responses need to be dampened when there is unwanted immunological activity (e.g. in autoimmunity or graft rejection).

## Subsets of Regulatory T Cells

### Naturally Arising Foxp3^+^ CD4^+^ Treg

Classically defined Treg are a subset of Foxp3 expressing CD4^+^ cells which maintain peripheral tolerance by suppressing autoreactive CD4^+^ cells that have escaped from negative selection in the thymus (7). Naturally arising Foxp3^+^ CD4^+^ Treg cells develop mostly in the thymus (tTreg) and require a relatively strong TCR signal which results in Treg cells having a repertoire enriched for self-antigen recognition (8). In the periphery, Treg can be generated upon TCR stimulation of naïve CD4^+^ Foxp3^-^ T cells in the presence of TGFβ and they are known as peripheral Treg (pTreg) (9). *In vitro*, Treg can also be produced from CD4^+^ Foxp3^-^ cells by mimicking *in vivo* conditions for pTreg generation (iTreg) (10). In contrast to tTreg, pTreg are likely generated upon exposure to non-self-antigens like allergens, food and microbiota (11).

It is important to keep in mind that whereas in mice Foxp3 expression is limited to Treg, many human Foxp3^+^ T cells are more similar to conventional T cells (Tconv) than to Treg, and some activated non-suppressive Tconv express low levels of Foxp3. Miyara and colleagues defined three different human T cell populations based on the expression of Foxp3 and CD45RA: Foxp3^low^ CD45RA^+^ as resting Treg; Foxp3^high^ CD45RA^-^ as activated/effector Treg, and Foxp3^low^ CD45RA^-^ as non-suppressive cytokine-producing non-Treg (12). Thus, it is fundamental to combine Foxp3 expression with other Treg markers (CD45RA, CD127 (IL-7R), CD25) in order to identify and analyze these cells in humans (13).

CD4^+^ Foxp3^+^ T cells can modulate immune reactions in a direct or indirect fashion. One of the most studied direct suppression mechanisms is the production of the anti-inflammatory cytokine IL-10, which can inhibit phagocyte function, antigen presentation, co-stimulatory molecule expression, T-cell proliferation, and impairs the production of IL-2 and IFNγ. Treg-produced IL-10 promotes tolerance in the intestinal mucosa and defects in IL-10 signaling trigger inflammatory bowel disease in mouse and human (14, 15). In contrast, IL-10 can stimulate NK cell activity, B cell activation and isotype switching (16). Transforming growth factor-beta 1 (TGFβ1) signaling is associated with the development, stability and function of Treg. TGFβ1 antagonizes negative selection in the thymus, supporting early Treg development (17). In the periphery, it is essential for the differentiation of Treg from naïve CD4^+^ Treg. TGFβ1 production by Treg and its autocrine signaling is required for Treg-mediated suppression, but several studies suggest that while it is not a major suppressor mechanism it might be needed under high inflammatory conditions (18). When Foxp3^+^ CD4^+^ Treg encounter effector T cells (Teff) and interact with them, one mechanism of suppression is the secretion of granzyme and perforin *via* exocytosis. By doing so, they can induce apoptosis in the target cells, e.g. in CD4^+^ CD25^-^ effector cells (19, 20).

Treg are able to indirectly turn down immune reactions by disturbing the optimal environment for immune responses by interfering with IL-2 availability, ATP/AMP balance, and the interface between T cells and DC. IL-2 is known for inducing and promoting T cell proliferation, but it also is involved in termination of T cell responses (21), since mice deficient in IL-2 or IL-2R suffer from a lymphoproliferative syndrome (22, 23). This negative effect on T cell activation happens indirectly by promoting the activation of anergic Treg, which then in turn suppress other T cells (24). Upon activation of naïve T cells, IL-2 is produced, which induces phosphorylation of STAT5 promoting Foxp3, Tbet and GATA3 expression and thereby the generation of Treg, Th1, or Th2 cells, respectively. At the same time, production of IL-17A and Bcl-6 and thereby differentiation towards Th17 or Tfh cells is inhibited (25, 26). Interestingly, high concentrations of IL-2 favor differentiation of effector T cells (27), whereas low IL-2 levels facilitate the production of memory T cell (28). Treg can interfere with these processes by modulating the amount of available IL-2. They suppress production of IL-2 by effector cells in a contact dependent manner *in vitro*. It has also been suggested that Treg can sense the source of IL-2 and migrate to the zones of immune activation where they “steal” IL-2 from other T cells promoting their apoptosis. This model remains controversial regarding *in vivo* studies since the source of IL-2 needs to be clarified (18).

Murine CD4^+^ Treg express high levels of the two ectonucleotidases CD39 and CD73 which can convert ATP into non-toxic AMP and AMP into the immune suppressive adenosine, respectively. In humans, co-expression of these ectonucleases is a rare event and most Treg express only CD39 which means they need to encounter CD73^+^ cells in order to produce adenosine (29). Extracellular adenosine binds the A2AR receptor expressed by Treg increasing their frequency and promoting their immune modulatory function (30). In the presence of excessive inflammation and tissue damage, there is an increase of extracellular ATP, which is cytotoxic for many cell types. Extracellular ATP promotes death signaling through P2X7R engagement in sensitive Treg while some Treg survive and convert into effector Th17 cells upon exposure to extracellular ATP (31). An additional strategy of Treg to escape apoptosis and survive in such environments is the conversion of ATP into non-toxic metabolites, such as AMP. In addition, AMP can indirectly alter the expression of proinflammatory cytokines and promote the expression of inhibitory ones. This translates into a reduction of costimulatory molecules in DC (32), less activation of effector cells and a higher suppressive capacity of Treg (30).

Treg can disrupt the microenvironment in the immunological synapse provided by DC which is essential for T cell proliferation. In detail, Treg act by either reducing the limiting enzyme for glutathione (GSH) synthesis or by consuming extracellular cysteine which is needed for T cell cycle progression and DNA synthesis (33).

Treg are also capable of removing surface molecules from antigen presenting cells (APC) during the immunological synapse. They can engulf part of DC membranes containing pMHCII and co-stimulatory molecules which leads to abrogation of T cell priming (34). Moreover, the inhibitory molecule CTLA-4, which is constitutively expressed on Treg has been described to remove CD80/CD86 from the surface of antigen presenting cells during the immunological synapse (35).

Some Treg have developed specialized adaptations to their environment. As an example, VAT-Treg (visceral adipose tissue-Treg) express high levels of PPARγ in order to reduce insulin resistance associated with inflammation of fat tissue (36).

### Non-Classical CD4^+^ Treg

As already mentioned above, there are other types of regulatory T cells that do not fit the phenotype of the classically defined CD4^+^ Foxp3^+^ Treg cells (**Table 1**). Many of them share the mechanisms of suppression of Treg described above, but some use different strategies.

**Table 1:** Different Treg subsets identified in human and/or mouse.

Cell population	Human	Mouse	Reference
CD4^+^ CD25^+^ Foxp3^+^	✓	✓	(5, 6)
CD4^+^ Foxp3^low^ CD45RA^+^	✓	✗	(12)
CD4^+^ Foxp3^high^ CD45RA^-^	✓	✗	(12)
CD4^+^ Foxp3^-^ IL10^+^ (Tr1)	✓	✓	(37)
CD4^+^ Foxp3^-^ TGFβ^+^ (Th3)	✓	✓	(38)
CD4^+^ Foxp3^-^ IL35^+^ (iTr35)	✓	✓	(39)
CD4^+^ Foxp3^-^ IL10^+^ TGFβ^+^ (Treg of B cells)	✗	✓	(40)
CD8^+^ Foxp3^+^ and/or CD28^-^ and/or CD25^+^	✓	✗	(41–43)
CD8^+^ CD45RA^+^ CCR7^+^ Foxp3^+^	✓	✗	(44)
CD8^+^ CD45RC^low/-^	✓	✓	(45)
CD8^+^ CD122^+^	✗	✓	(46)
CD8^+^ HLA-DR^+^	✓	✗	(47)
CD8^+^ HLA-E^+^	✓	✗	(48)
CD8^+^ Qa-1^+^	✗	✓	(48)
γδ T cells	✓	✓	(49)
NKT	✓	✓	(50)

Treg cells are categorized based on CD4 or CD8 surface markers expression.

Type 1 regulatory T cells (Tr1) are a population of CD4^+^ Foxp3^-^ cells expressing high levels of the anti-inflammatory cytokine IL-10. They are generated in the mucosa-associated lymphoid tissue (MALT) when naïve CD4^+^ cells encounter IL-10 produced by APC. Tr1 cells control T cell responses in infection and autoimmunity and they have been shown to produce higher levels of IL-10 than Foxp3^+^ Treg (37). Another subset of adaptive Treg are the Th3 cells, which are a unique population of T helper cells induced by oral tolerance to non-self-antigens. Th3 cells produce high concentrations of TGFβ and moderate amounts of IL-10 (38). In contrast to Tr1 cells which do not express Foxp3, some Th3 cells are TGFβ-induced-Foxp3^+^ cells (51). Collison and colleagues described another inducible type of Treg (iTr35) which do not secrete IL-10 or TGFβ, but instead IL-35, an inhibitory member of the IL-12 pro-inflammatory family. This cytokine suppresses T cell responses and expands Treg by inducing the conversion of conventional T cells into suppressive Foxp3^-^ regulatory T cells (iTr35). iTr35 cells are highly suppressive and stable *in vivo*, they are key mediators of infectious tolerance and can contribute to Treg-mediated tumor progression (39).

Similar to other components of the immune system, B cells are involved in the expansion and generation of Treg. In the periphery, naïve B cells can convert CD4^+^ CD25^-^ cells into CD4^+^ CD25^+^ Foxp3^-^ Treg (Treg-of-B cells) in a cell-cell contact dependent manner. These Treg-of-B cells express molecules characteristic for Treg, such as IL-10, TGFβ, CTLA-4, PD-1, LAG-3, GITR, ICOS, and OX40. They exert their suppressive function *in vivo* and *in vitro* in antigen-specific and antigen-independent manners, utilizing IL-10-mediated as well as other suppressive mechanisms (40).

### CD8^+^ Regulatory T Cells

CD8^+^ T cells were first described to exert immunosuppressive functions more than 40 years ago, but the lack of specific markers and therefore the difficulties in isolating this population limited this area of research (52). Interest in CD8^+^ Treg cells increased again after the revival of the concept of T-cell-mediated immunosuppression in the mid-1990s and the study of CD4^+^ Treg cells. In the following years the immunosuppressive properties of CD8^+^ cells were independently characterized by several groups (53).

Foxp3 is preferentially expressed in CD4^+^ CD25^+^ cells in mice and it is barely detectable in CD8^+^ T cells. The expression of the transcription factor HELIOS or the surface marker CD122 or the lack of CD28 on the surface are used to identify CD8^+^ Treg in mice (46). In contrast, human CD4^+^ as well as CD8^+^ T cells are able to express Foxp3, but levels of this transcription factor are substantially higher in CD4^+^ compared to CD8^+^ cells (54). In contrast to CD4^+^ regulatory T cells, there is no reliable marker to define CD8^+^ Treg cells, but they are rather identified by their immunosuppressive function.

Firstly isolated from rats, CD8^+^ CD45C^low/-^ Treg cells suppress the proliferation of CD4^+^ T cells and their differentiation into a Th1 phenotype. They produce IL-4, IL-10, and IL-13 and express CTLA-4 and Foxp3 (45). Sorted human IFNγ^+^ IL‐10^+^ CD8^+^ CD45RC^low/‐^ Treg are more potent suppressor cells than the rest of the CD8^+^ CD45RC^low/‐^ Treg and blockage of IFNγ abrogated their suppressive activity in a model of allogenic cardiac transplantation (55).

Foxp3^+^ CD8^+^ cells are rarely detected in human blood but they are found in human tonsils where they express high levels of CTLA-4 and CD45RO but only little CD127 and CD69. Tonsillar Foxp3^+^ CD8^+^ Treg are CD25^-^ and express pro-inflammatory cytokines like TNFα, IFNγ, and IL-17 (41). Foxp3 expressing CD8^+^ T cells have also been found in blood of HIV-infected individuals showing an activated (HLA-DR, Ki-67, and PD-1 expression) and senescent (CD57^+^ CD28^-^) phenotype (56). Several studies show that CD8^+^ Foxp3^+^ Treg can be induced under certain conditions. *In vitro* stimulation of peripheral blood mononuclear cells (PBMC) with anti-CD3 mAb has been shown to induce CD8^+^ CD25^+^ Foxp3^+^ T cells, which were able to suppress proliferative responses to *Staphylococcal* enterotoxin B (SEB), and that the inhibitory effect was partially depending on CCL-4, TNF, and IL-2 (43). CD8^+^ CD28^-^ Foxp3^+^ cells can be generated *in vitro* after multiple rounds of stimulation of PBMC with allogenic or xenogenic APC. They are believed to tolerize APC by up-regulating the inhibitory receptors immunoglobulin-like transcript 3 (ILT-3) and 4 (ILT-4) and down-regulating costimulatory molecules such as CD58 and CD86 (42). Upon suboptimal TCR stimulation in the presence of IL-15, CD8^+^ CCR7^+^ T cells express Foxp3 (they become CD8^+^ CD45RA^+^ CCR7^+^ Foxp3^+^) and acquire immunosuppressive functions. They prevent CD4^+^ T cells from responding to TCR stimulation by directly interfering with the TCR signalling cascade and not by the usual suppression mechanisms mediated by IL-10, TGFβ, or CTLA-4 (44). In addition, these CD8^+^ Treg release exosomes carrying NADPH oxidase 2 (NOX2), which are taken up by CD4^+^ T cells and inhibit their proliferation *in vivo* and *in vitro* (57).

Most studies investigating non-Foxp3 expressing CD8^+^ Treg in humans describe them as CD8^+^ CD28^-^ cells, although CD8^+^ CD28^+^ Treg can be generated *in vitro*. Mechanistically, CD8^+^ CD28^-^ Treg act by i) influencing CD80/CD86 surface expression of DC leading to inhibition of CD4^+^ T cell responses, or ii) secretion of IFNγ and IL-6 cytokines or iii) secretion of the anti-inflammatory cytokine IL-10 (54). In addition, CD8^+^ CD28^-^ Treg express high levels of the IL-2 receptor CD122 and this has been used as a marker for the characterization of these CD8^+^ CD28^-^ Treg. On the other hand, a population of CD8^+^ CD28^+^ Treg cells expressing the chemokine receptor CXCR5 has been identified, which are capable of suppressing B cell responses and antibody production by inhibiting follicular helper T (Tfh) cell-mediated B cell differentiation (58). They also exert strong antitumor activity and their presence is associated with favorable prognosis in follicular lymphoma patients (59).

In mice and humans, CD8^+^ Treg have been described to preferentially recognize the non‐classical MHC class I molecules Qa‐1 (mouse) or HLA‐E (human) which are orthologous genes. These non‐classical MHC class I restricted populations have the property to recognize TCR, MHC or heat shock protein derived peptides (i.e. Qdm, HSP60sp) presented by Qa‐1 or HLA‐E (48). CD8^+^ Treg exert a cytotoxic effect against antigen−activated CD4^+^ T cells, and this function depends on the expression of the MHC−Ib molecule Qa−1 in mice (54).

Whereas CD8^+^ CD45RO^+^ CCR7^+^ T cells are found in blood and have no suppressive function, CD8^+^ CD45RO^+^ CCR7^+^ IL-10^+^ suppressive cells are found intratumorally in ovarian cancer patients and they are believed to be induced by plasmacytoid DC (60).

In 2014, a novel population of CD8^+^ Treg characterized by the expression of HLA-DR was identified in human peripheral blood and umbilical cord blood. CD8^+^ HLA-DR^+^ cells suppress in a cell-to cell contact dependent manner, which involves CTLA-4 (47). Within the CD8^+^ HLA-DR^+^ Treg cells the CD28^+^ subpopulation shows higher suppressive capacity compared to their CD28^-^ counterparts and also expresses higher levels of the checkpoint inhibitory molecules CTLA-4, TIM-3, PD-1 and LAG-3 (61). Similarities have been found between CD8^+^ HLA-DR^+^ and CD4^+^ Foxp3^+^ Treg with regards to the expression of TIGIT, the chemokine receptors CCR4 and CCR5, the low expression of IL-7R (CD127) and a memory and effector-like phenotype. In addition, after polyclonal TCR stimulation, CD8^+^ HLA-DR^+^ Treg cells increase IFNγ and TNFα expression suggesting that they are not exhausted cells despite the fact that they express PD-1 (62).

### CD4^−^CD8^−^ Regulatory T Cells

Gamma-delta T cells (γδT) are the first T cells to develop in the thymus upon gene rearrangement which generates different TCR chains (γδ) than the more abundant αβT cells during fetal ontogeny. In contrast to αβT cells, γδT cells do not undergo thymic TCR selection (63). γδT cells represent a small T cell population (3–5% in human peripheral blood) and are able to interact with different immune cell types such as other T cells, B cells, DC, NK cells, monocytes/macrophages, and granulocytes. In some cases, they exert an anti-inflammatory effect. These regulatory γδT cells have been studied in different contexts and are associated with immunosuppression in pregnancy, inflammation, allergy and cancer. Similarly to αβT cells, γδT cells produce TGFβ and IL-10 together with variable expression of the transcription factor Foxp3 (49).

Natural killer T (NKT) cells are a special subset of T cells that co-express NK cell surface receptors (NK1.1/CD161) with the semi-invariant T-cell receptors (TCR), which consist of an invariant TCR α chain paired to a limited number of TCR β chains. Like all T cells, they are generated in the thymus, but most of the NKT cells do not express CD4 or CD8 on their surface (64). Upon activation, NKT cells produce large amounts of Th1 (including IFNγ and TNFα) and Th2 (IL-4, IL-10, and IL-13) cytokines enabling them to act as powerful regulators of the immune system (50). Under certain conditions, NKT cells can exert potent suppressor functions by shifting from Th1 to Th2 responses both in human and mouse. Their main target cells of suppression are tumor cells, pathogen-activated T cells and APCs (65).

## Roles of Treg in Different Scenarios

Treg constitute a group of phenotypically distinct subsets that can reside in lymphoid and in non-lymphoid organs where they exert diverse functions. The main non-lymphoid tissues where Treg can be found are the visceral adipose tissue (VAT), the intestine, skin and muscle. In these four tissues Treg are important regulators of inflammation and fibrosis and contribute to tissue repair (66). As already mentioned, Treg participate in numerous processes in which they adapt to the environment in order to eventually maintain tissue homeostasis. Some situations such as pregnancy, organ transplants or the common presence of bacteria in the intestine, require tolerance against foreign antigens for which Treg activity is essential. If Treg activity is too low, there can be a failure in self-tolerance leading to the development of autoimmune diseases. On the other hand, it can be hypothesized that if Treg are over-active, they may favor the progression of neoplasic malignancies. During pregnancy, the allogenic nature of the fetus (harboring maternal and paternal antigens) requires tolerance from the mother’s immune system in order not to be rejected. Treg and other immune cells create a tolerogenic environment whose composition changes throughout gestation. At least five different Foxp3^+^ Treg subtypes have been identified during different stages of pregnancy. In addition, other minor Treg subsets such as CD4^+^ HLA-G^+^ Foxp3^-^ (which inhibit NK, CD4^+^ and CD8^+^ T cells), Tr1, Th3, γδT cells, TIGIT^+^ T cells, and CD8^+^ Treg have been detected in the decidua and/or peripheral blood of pregnant women (67).

Barrier tissues are constantly exposed to dietary, environmental, and commensal microbiota antigens and therefore immune homeostasis and tolerance need to be ensured *via* Treg or Teff antigen-specific repertoires in these tissues (68). In intestinal tissues, this tolerance is achieved by the cooperation of different immune populations including Foxp3^+^ Treg and Tr1 cells. Dysregulated intestinal responses to dietary antigens or commensal microbiota frequently lead to immunological disorders in humans such as celiac disease, food allergy, and inflammatory bowel disease (69). Encounter with commensal microbiota generates pTreg rather than anti-microbial effector cells. These pTreg use site-specific TCRs different than the ones that facilitate tTreg development in the thymus, implying that many colonic Treg arise by means of antigen-specific driven pTreg development (70).

Several reports highlight Treg functional deficiencies in autoimmune diseases, but the underlying molecular mechanisms are still unknown. One of the major limitations of studying human autoimmunity is the lack of validated experimental assays and the discrepancies between *in vitro* and *in vivo* experiments (71). Also, there is little consensus for Treg identification (i.e. agreement on the markers used for their identification), which makes the comparison between different studies almost impossible (13). Treg dysfunction in autoimmune diseases can be grouped according to different factors (68): i) Genetic disease like germline mutations in the Foxp3 locus. The development of the severe immune dysregulation, polyendocrinopathy, enteropathy, and X-linked (IPEX) syndrome is due to point mutations and microdeletions in the Foxp3 gene that impair Treg function (72). ii) The abrogation of Treg promoting signals. The disruption of the IL-2/IL-2R pathway dysregulates thymic development and peripheral homeostasis of Treg. In a murine model of Type 1 diabetes, pancreatic Treg die showing a decreased expression of the IL-2R CD25 and of the anti-apoptotic protein Bcl-2 (73). iii) The presence of Treg destabilizing factors. Overexpression of IL-6 and TNFα can interfere with Foxp3 expression and consequently alter Treg/Teff balance. In the presence of TGFβ, IL-6 enhances RORγt expression, which induces Th17 generation *via* STAT-3, and represses at the same time Foxp3 expression. In the case of rheumatoid arthritis, high IL-6 levels are related to a preferential development of Th17 cells over Treg in the periphery (74, 75).

In addition to maintaining immune homeostasis in the lymphoid tissues, Treg are recognized as regulators of non-immunological processes. Treg are present in healthy tissues and upon tissue injury, they promote tissue regeneration in an amphiregulin-dependent manner (76). In a model of influenza virus infection, Treg-induced tissue repair is triggered in response to the inflammatory mediators IL-18 and IL-33, but not by TCR signaling, which is required for their suppressive function (77).

Increased immune suppression contributes to cancer onset and tumors promote the generation of an environment that allows tumor cells to escape from immune responses. Cancer cells develop immunosuppressive mechanisms such as expression of anti-inflammatory mediators and recruitment of suppressive leukocytes, such as Treg, myeloid derived suppressor cells (MDSC), tolerogenic DC and tumor-associated macrophages (TAMs) (68). In recent years it has been found that many tumors are enriched in Treg cells, indeed, a high Treg/Teff ratio correlates with poor prognosis (78). Treg may facilitate cancer progression *via* suppression of effector cells that otherwise would attack the tumor. Turnis and colleagues showed enrichment of IL-35-secreting Treg cells in tumors and demonstrated that Treg-derived IL-35 promotes T cell exhaustion in the tumor microenvironment (79). In fact, different tumor models benefit from Treg depletion leading to an increased anti-tumor response (80–82), but not all tumors benefit from the presence of Treg cells. In addition, CD4^+^ CD25^+^ Treg collaborate with CD8^+^ CD28^-^ Treg cells within different tumors so that the immunosuppressive activity of these tumor infiltrating Treg cells may be predominant (83). In multiple myeloma patients, CD8^+^ CD57^+^ lymphocytes show an activated phenotype (HLA-DR^+^ and Fas^+^) and can inhibit the suppressive effect of Treg as well as antibody production (84). However, the effect of Treg depends on the tumor site, molecular subtype and tumor stage (85). Interestingly, Foxp3^+^ tumor infiltrating Treg were associated with better prognosis in colorectal cancer (86). These cells were later shown to not be fully suppressive and to display some inflammatory T cell features (87). In human follicular lymphoma, high amounts of intratumoral Treg were related to positive outcome (88, 89) whereas high levels of circulating Treg correlated with a negative prognosis (90). All these discrepancies indicate that the presence of intratumoral or circulating Treg may depend on the nature of the tumor, the tumor microenvironment itself and the functionality of the supposedly suppressive Treg.

Low responsiveness and reduced proliferation of virus-specific T cells during chronic viral infection is associated with the expansion of Treg. In acute and chronic murine retroviral infection models, depletion of Treg decreases viral load and restores the activity of virus-specific cytotoxic CD8^+^ T cells (91, 92). On the other hand, it has been found that the role of Treg depends on the disease stage in tuberculosis patients. Treg expand and delay immune responses in initial phases but they counter-regulate excessive inflammation later in the chronic phase (93). In a mouse model of chronic infection with Friend retrovirus (FV), vaccination with a calcium phosphate nanoparticle-adjuvant, which efficiently reactivated CD8^+^ T cells, in combination with a transient ablation of Treg enhances anti-viral immunity (94).

Germinal centers (GC) are transient structures in peripheral lymphoid organs where B cells develop and differentiate into antibody secreting plasma and memory B cells. Upon activation by follicular dendritic cells, B cells proliferate and interact with primed antigen-specific Tfh cells in order to be fully activated and differentiate into antibody-secreting plasmablasts (95). Recently, a new Treg subset has been described in GC called follicular regulatory T cells (Tfr). These Tfr cells control Tfh-driven GC responses preventing induction of autoreactive and foreign antigen-specific antibodies (96). They control IgG and IgE responses to vaccines, allergens and autoantigens, and have a critical immunoregulatory function before GC formation (97). The relevance of these Tfr in the control of antibody production has drawn the attention of several studies which investigate these cells as new targets for immunotherapy.

## Treg Homeostasis and Function in Aging

The severity of many infections increases with age and many of the vaccines currently used are less effective in older compared to younger adults. This is due to changes that the immune system undergoes over time leading to dysregulation the adaptive and, to a lesser extent, innate mechanisms (**Table 2**). In old age, the capacity of APC to process and present antigens to T cells declines (98) and chemotaxis, cytokine production and signal transduction upon antigen recognition are impaired in these cells as well as in neutrophils (99, 100). In addition, B cells show reduced somatic hypermutation and class switch and the number of plasma cells is decreased which leads to lower antibody production (101). The T cell compartment is skewed towards effector/memory like T cells, shows a reduced TCR repertoire and also accumulates DNA damage in aged individuals (102). In this review, we aim to describe the processes relevant for Treg, but extensive reviews on general immunosenescence can be found elsewhere (103–105).

**Table 2:** Immunological changes during aging.

Immune organ/cell	Changes in aging
Thymus	↓ bone marrow progenitors
	↓ sex hormones
	↓ thymic ephitelial cells
	↑ adipose tissue
APC	↓ antigen processing capacity
	↓ antigen presenting capacity
	↓ chemotaxis, cytokine production and signal transduction
Neutrophils	↓ chemotaxis, cytokine production and signal transduction
B cells	↓ somatic hypermutation
	↓ Ig class switch
	↓ number of plasma cells
T cells	↓ TCR repertoire
	↑ effector-memory cells
	↑ DNA damage accumulation

A summary of the most prominent changes in the thymus as well as in different immune cells is described according to their main function.

During aging, the thymus undergoes a gradual reduction in size and function together with changes in its architecture (106). The activity of key factors for thymus functionality is also modified. Less bone marrow progenitors reach the thymus, the flow of sex hormones is diminished, thymic epithelial cell (TEC) number decreases and there is an increase in adipose tissue (107). TEC are part of the structural environment necessary to support the normal differentiation of thymocytes. TEC-mediated thymic involution results in reduced cellularity capable of maintaining normal thymocyte differentiation (108). Despite the decline in thymic cellularity, no blockade of thymocyte differentiation is observed and thymic function is maintained proportional to the reduced size (109). Adipocytes are not only responsible for anatomical changes within the thymus but they actively contribute to thymic involution. They produce higher levels of negative factors for thymic maintenance (e.g. IL-6, sex hormones and steroids) and thereby transmit suppressive signals to TEC reducing thymopoiesis and cellularity (107). During aging, the overall endocrine profile changes with an extra-thymic reduction of sex hormones and growth hormones, and an increase in glucocorticoid levels (110). Estriol and chorionic gonadotropin positively affect Foxp3 expression and increase Treg frequency. Steroids and glucocorticoids can enlarge Treg populations in the periphery stimulating their function (111). In fact, Treg lacking the glucocorticoid receptor lose their suppressive function turning into Th1-like, IFNγ-producing cells in a murine colitis model (112).

In the periphery, the number of recent thymic emigrants (RTE) is decreased in aged mice and humans which implies a lower contribution of thymic cells to the pool of total T cells in the body (113–115). In mice, it has been observed that the production of Treg cells declines more and faster than conventional T cell production. This is attributed to the inhibitory effect of recirculating Treg cells on the differentiation of new Treg when the former revisit the thymus (116). This accumulation of antigen-specific Treg reduces clonal diversity which translates into a skewed aged Treg pool which can suppress only certain T cells while leaving the rest unaffected. This might favor some proinflammatory cells to remain active in aged hosts (111).

Aging and age-related diseases are associated with profound changes in epigenetic patterns. Treg are subject of epigenetic modifications that are altered during aging. It has been shown that hypomethylation of CpG sites upstream of the Foxp3 enhancer correlates with a higher suppressive function of Treg from aged mice (117).

In addition, pTreg differentiation also decreases with age. It has been observed that naive conventional T cells from aged mice differentiate less into pTreg *in vivo* and *in vitro* compared to their young counterparts (118). Despite less thymic and peripheral Treg differentiation, Treg accumulate with age, which could be explained by the loss of the pro-apoptotic protein Bim rendering these cells apoptosis-resistant compared to Treg from young individuals (119). Loss of Bim or overexpression of Bcl-2 lead to Treg accumulation, but at the same time to reduced suppressive capacity in a model of murine colitis (120). On the other hand, *in vitro* studies show that Treg from young and old adults have the same suppressive capacity (121). Others describe that old Treg are better suppressors than Treg from young adult mice due to a higher IL-10 production from the older ones (117). Despite many discrepancies among different studies, the overall data suggests that Treg function remains unchanged or is even enhanced in the elderly. Aged individuals are more prone to develop infections and neoplastic malignancies which would agree with enhanced Treg function, whereas they are also more susceptible to develop autoimmunity due to Treg dysfunction (122).

In the periphery, total T cell numbers remain unchanged with age maintaining an adequate pool of circulating T cells including Treg. In peripheral blood mononuclear cells (PBMC) from aged humans an increased proportion of activated Treg (Foxp3^hi^ CD45RA^-^) and a low but detectable resting Treg population (Foxp3^low^ CD45RA^+^) are present. Both populations show suppressive potential but the activated Treg die after exerting *in vitro* suppression (12). It has been observed that there is an accumulation of CD25^low^ Foxp3^+^ Treg in old age and this correlates with lower IL-2 levels. The IL-2 receptor (IL-2R), consist of the subunits IL-2Rα (CD25), IL-2Rβ (CD122) and the common gamma chain (CD132), and is expressed on T and B cells, DC and steady state NK cells. CD25^low^ Foxp3^+^ Treg upregulate the surface molecule CD122, which is also part of the IL-15 receptor, suggesting that IL-15 might support CD25^low^ Treg in old age (123). Tr1 cells also accumulate in aged mice in an IL-6 dependent manner and are able to produce large amounts of IL-10 (37). Follow-up studies show that these IL-10 producing cells manifest a Tfh phenotype and that they are involved in immune suppression in old age (124). Van der Geest and colleagues studied different CD4^+^ T cell populations in healthy individuals and observed that the proportion of naive Treg cells declined with age whereas the memory Treg compartment and the memory Treg/Teff ratio increased. This accumulation of memory Treg in old age is associated with poor responses to influenza vaccination (125). Tfh cells from aged mice express high levels if IFNγ and IL-10 and an environmental increase in TGFβ levels in old mice favors the development of Treg (126). After vaccination in old age, impaired differentiation of Tfh cells followed by a suboptimal T cell priming is observed with a consequent poor GC B cell expansion. This is thought to be due to not only lower T cell activation but also to an accumulation of Treg which negatively affect the GC reaction (127). Along these lines, another study in aged patients showed that non-responder individuals to influenza vaccination have a higher frequency of Treg as well as a higher inflammatory status compared to responders (128). Depletion of Treg with anti-CD25 treatment prior to influenza infection protected against lethal viral challenge in aged mice (129). In another study, responders to influenza vaccination showed a higher frequency of Treg compared to non-responders, together with an elevated frequency of early differentiated CD4^+^ T cells and lower proportion of memory CD4^+^ cells (130). Actually, a detailed study in the context of inflammation showed that, upon secondary activation, memory Treg could not undergo pronounced recall expansion as conventional CD4^+^ T cells do (131). These findings emphasize the importance of the accumulation of memory Treg during aging and their role in the ineffectiveness of vaccination in the elderly.

Similar to CD4^+^ cells, the CD8^+^ T cell pool also loses naïve precursor cells but the contraction of the naïve CD8^+^ T compartment is greater than the one for CD4^+^ T cells (132). In human and mouse, naturally occurring CD8^+^ Foxp3^+^ Treg numbers increase with age (133, 134) whereas inducible CD8^+^ CD45RA^+^ CCR7^+^ human (135) and CD8^+^ CD44^+^ CD62L^+^ CCR7^+^ mouse (43) Treg cells decline in old age. The increase of CD8^+^ Foxp3^+^ CD28^–^ Treg is consistent with the higher overall numbers of CD8^+^ CD28^–^ T cells in aged individuals (136). Despite the fact that CD8^+^ HLA-DR^+^ Treg accumulate with age, the expression of CD28 in these cells remains unchanged. CD8^+^ HLA-DR^+^ Treg cells lose suppressive activity and decrease the production of checkpoint inhibitory molecules in aged individuals (61).

Together with CD4^+^ CD25^+^ Treg frequencies observed in the elderly, CD8^+^ Treg accumulation has been suggested to contribute to the decline in adaptive immune responses in the aging process (137). The capability of human CD8^+^ CCR7^+^ cells to differentiate into Foxp3-expressing cells negatively correlates with age (44). It has been shown that expression levels of Foxp3 and CD45RA in CD8^+^ CCR7^+^ Treg are lower in older individuals compared to young, and this may suggest a diminished suppressive capacity of these cells potentially contributing to the development of autoimmune diseases in the elderly (138). In contrast, the ability of aged naturally occurring CD8^+^ Treg to suppress the proliferation and cytokine production of effector CD4^+^ T cells remains similar between younger and elder individuals (137). Dysfunction of CD8^+^ and CD4^+^ Treg cells in old age could support age-associated subclinical inflammation referred to as “inflamm-aging” (139, 140), which is associated with increased levels of oxygen radicals, IL-15, TNFα and IFNγ (141, 142).

**Figure 1:**
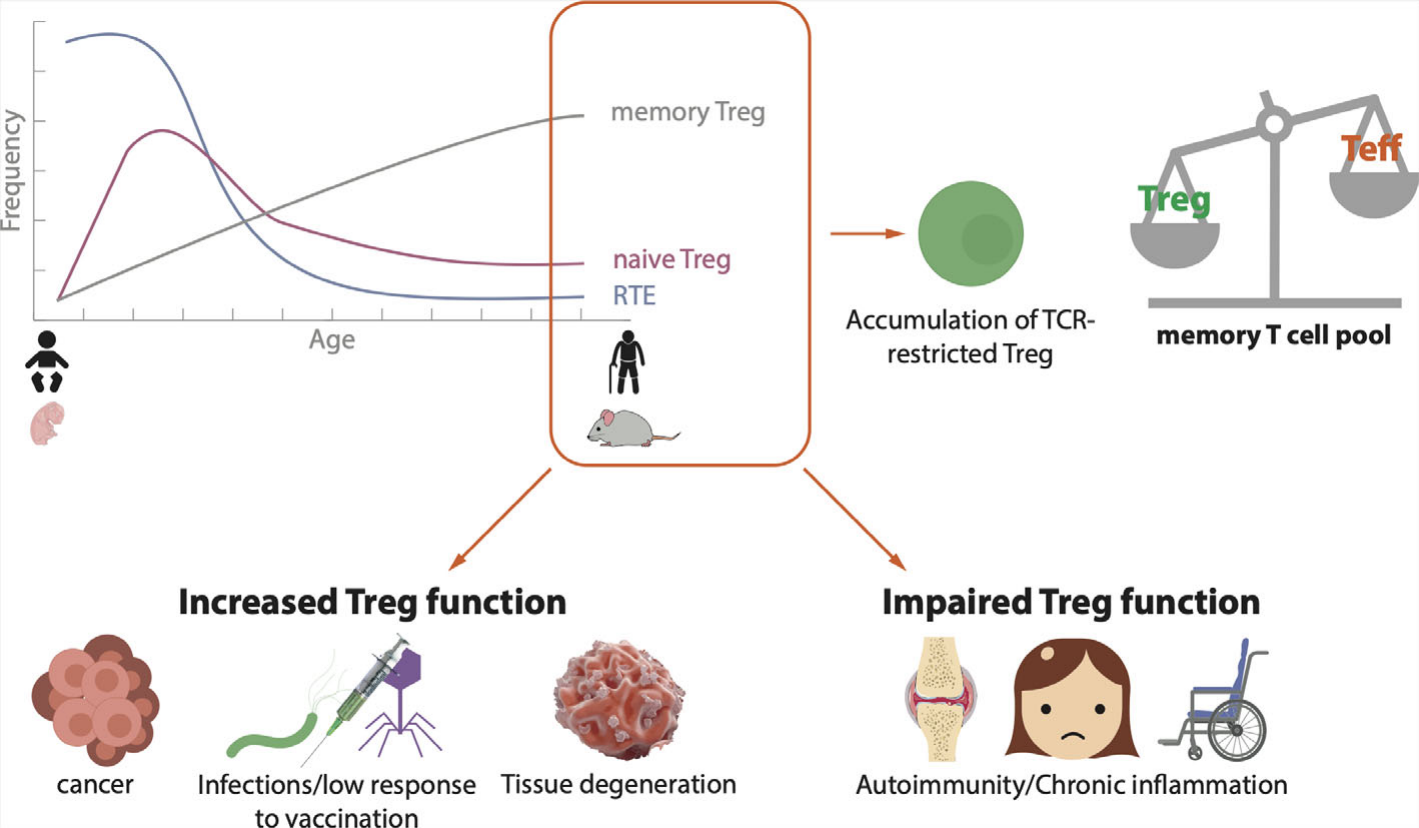
Schematic landscape of Treg in the elderly. During human and mouse aging, there is a decreased efflux of recent thymic emigrants (RTE) to the periphery, a reduction of naïve Treg and an accumulation of antigen experienced memory Treg. As a consequence, TCR-restricted Treg accumulate and the pool of memory T cells is skewed towards memory Treg over effector/memory T cells. This imbalance on T cell homeostasis is related to the development of different diseases. In old age, an increase in Treg function may lead to the progression of tumors, chronic infections or tissue degeneration, whereas an impaired Treg function may cause autoimmunity and/or chronic inflammation.

Taken together these data suggest that CD4^+^ Treg as well as CD8^+^ Treg accumulate in aged individuals. Dysregulation of immune homeostasis is involved in the development of different diseases in old age and a connection with increased numbers of Treg has been demonstrated for several conditions (**Figure 1**).

## Discussion

The findings discussed above show that the field of regulatory cells is in continuous expansion. The more we try to define it, the more complex becomes the diversity of different cells (T, B, and NKT cells) that might be involved. Initially described as suppressor cells, Treg have demonstrated to be able to regulate several processes with the final aim of preserving immune homeostasis. To date, there are numerous types of Treg described in mouse and human (**Table 1**) and a lack of consensus to establish the right markers to define these populations make of their characterization a matter of controversy. The promiscuity of Foxp3 expression as a *bona fide* marker of Treg is a good starting point to discuss the huge diversity of Treg identified so far. There is not a single marker distinct for Treg but a combination of some of them (Foxp3, CD25; GITR; CTLA-4, IL-10, etc) together with their suppressive activity could help to identify this elusive type of cells. Furthermore, most of the studies on human Treg are based on circulating cells in the blood, which may not represent the real landscape in a given tissue.

Treg cells are extremely versatile. They exert various effector functions and use a number of molecular mechanisms depending on the tissue and the health or disease context. Dysregulation of Treg function in one or another direction can unchain a huge variety of ailments (**Figure 1**). Indeed, modulation of Treg activity is a main target for anti-tumor therapies, treatment of autoimmunity or the avoidance of graft rejection in transplantation, for instance. The danger of targeting Treg to treat or prevent a certain disease is that the consequence could promote the development of another one. As an example, therapies for autoimmune diseases, which enhance Treg activity in order to dampen the autoreactive immune components, have the potential to increase the risk of tumor development as the suppressed immune cells might be unresponsive to malignant cells.

The level of complexity increases further when we add the factor age. In the elderly, the immune compartment is altered due to thymic involution and dysregulation of several immune processes. Most of the studies show that there is an accumulation of antigen-experienced Treg with age, but discrepancies exist regarding the hypo- or hyper-activity of these cells. This difference in Treg accumulation may not only be age-dependent but also context-dependent (**Figure 1**). In some cases, this results in increased responsiveness of aged individuals to Treg targeted therapies compared to young ones, as it is the case for anti-PD1 therapy in melanoma patients (143).

The vast diversity of Treg in different tissues and the lack of molecular markers to clearly define Treg subtypes makes this area of study highly complex. The clear increase in immune regulatory cells in aging is offering the scientific community plenty of opportunities to learn the context-specific roles of Treg cells in immunosenescence, advancing the concept into a more tailored tissue/disease/Treg-specific point of view.

## Author Contributions

LRR, FLM, RW, and BW wrote and discussed the manuscript, and all authors agree to be accountable for the content of the work. All authors contributed to the article and approved the submitted version.

## Funding

This work was supported by funds of the Austrian Science Fund (FWF W1253-B24; doctoral programme HOROS).

## Conflict of Interest

The authors declare that the research was conducted in the absence of any commercial or financial relationships that could be construed as a potential conflict of interest.
